# Perceptions of Lesotho nurse-midwives regarding post-partum depression management

**DOI:** 10.4102/curationis.v47i1.2624

**Published:** 2024-12-18

**Authors:** Ntsehiseng Maloleka, Libuseng M. Rathobei, Nellie Naranjee

**Affiliations:** 1 Department of Nursing, Faculty of Health Science, National University of Lesotho, Maseru, Lesotho; 2 Department of Nursing, Faculty of Health Sciences, Durban University of Technology, Durban, South Africa

**Keywords:** perceptions, nurse, midwives, post-partum depression, maternity ward, management

## Abstract

**Background:**

Effective post-partum maternal care is essential for the overall well-being of both the mother and the child. Postpartum depression (PPD) is a pervasive issue with profound implications for maternal health. However, a significant research gap exists concerning the perspectives of nurse-midwives on PPD within the context of Lesotho.

**Objectives:**

This study aimed to explore perceptions of nurse-midwives about PPD management at a tertiary care facility in Lesotho.

**Method:**

The study site was the Quthing Hospital, a government-funded healthcare facility situated in Lesotho’s southernmost district, Quthing. Employing a constructivist paradigm, the research adopted a qualitative, exploratory, descriptive and contextual design. Using purposive sampling, nine nurse-midwives participated in individual interviews.

**Results:**

Thematic analysis of the data resulted in three themes emerging namely: a lack of nurse midwifery empowerment, inadequate human and material resources and stigma on mental health issues.

**Conclusion:**

Nurse-midwives at a large tertiary care facility perceive PPD management through a multifaceted lens. Insights underscored the complexity of PPD and its ramifications for maternal care.

**Contribution:**

This study provides invaluable perspectives from nurse-midwives within a specific Lesotho context, laying the foundation for strategies to enhance PPD management and maternal mental healthcare.

## Introduction

Nurses are vital to the healthcare system, especially in maternal and child health, as the first point of contact for postpartum women seeking care, their knowledge and attitude towards postpartum depression (PPD) are critically important (Arefadib, Shafiei & Cooklin 2023). Postpartum depression is a widespread mental health problem and one of the prime causes of maternal suffering and ill-health. On a global level, the prevalence of the disorder is about 10%–15% (Agrawal, Mehendale & Malhotra 2022). In the United States, approximately 13.2% of PPD women were diagnosed with PPD, whereas in Georgia, the percentage of women diagnosed with PPD was slightly higher according to the last America’s Health Ranking (AHR) report in 2019 at 14.1% (America’s Health Ranking 2021). The study conducted in South Wales, United Kingdom revealed that majority of midwives had cared for postpartum depressed women (87.0%, *n* = 127) and had assessed women’s mental health informally by observing or asking questions about mood (99.3%, *n* = 144), anxiety levels (94.5%, *n* = 137), level of support (91.0%, *n* = 132) and mental health history (95.9%, *n* = 139) (Savory, Sanders & Hannigan 2022). In addition, the study conducted in the United States, revealed that screening for mental health problems including suicide during perinatal period is critical in efforts to help prevent depression (Chin et al. 2022). Programmes such as Massachusetts Child Psychiatry Access Project (MCPAP) for Moms and Making Access to Treatment, Evaluation, Resources and Screening better (MATTERS) used in North Carolina are perceived by nurses as helping build primary care and obstetric provider’s capacity to treat perinatal depression through education, telephonic perinatal psychiatric consultation and care coordination (Kimmel 2020). However, the study conducted in Australia revealed that nurse-midwives perceived that the complex system of operation (referral power, availability and access to mental support, a lack of sufficient time for adequate support) was a barrier to respond to women with PPD (Arefadib et al. 2023).

Similarly, Nweke et al. (2024) state that PPD is a significant public health concern in resource-constrained sub-Saharan Africa (SSA). The prevalence of postpartum depressive symptoms ranged from 3.8% to 69.9% in SSA. South Africa (30.6%) and Zimbabwe (29.3%) reported the highest prevalence rates, while Tanzania (13.5%) reported the lowest prevalence estimates. Postpartum depression constitutes a significant health burden in SSA and is fast becoming an epidemic in southern Africa. Consequently, Alba (2021) asserted that hospitalisation of pregnant women gives nurses the chance to educate mothers on PPD and other mood disorders; hence, nurses’ knowledge is of importance in mental health. A study, conducted in Ghana by Anokye et al. (2018), revealed that most common interventions used in management of PPD among respondents were psychosocial support (34%), professionally based postpartum home visits (28%) and interpersonal psychotherapy (20%) and cognitive therapy (18%). A study conducted in Nigeria showed that majority of respondents sometimes or never screened women for PPD (91.1%, *N* = 184), while 8.9% (*n* = 18) respondents always or often screened women for PPD and 87.0% (*n* = 176) had good attitudes towards screening for PPD (Mohammed-Durosinlorun, Mamoon & Yakasai 2022).

South Africa has a high prevalence of PPD with studies (Atuhaire et al. 2020; Nweke et al. 2024) reporting 30% – 35% of women diagnosed with major depressive disorders in the postpartum period. Additionally, in Cape Town, South Africa, nurse-midwives were more concerned about the time it took to assess women with PPD, and their heavy workloads and large patient numbers allowed them limited time with the patients (Abrahams et al. 2023). However, in Lesotho, there is no evidence-based research regarding the perceptions of nurse-midwives regarding PPD; hence, the researcher sought to explore perceptions of nurse-midwives regarding management of PPD at Quthing, Lesotho.

Postpartum depression is classified as one of the major psychological disorders that affect both the mother and baby. Frequently underdiagnosed, it remains the most common complication of childbirth and the most common perinatal psychiatric disorder, with women at the greatest risk during their first postpartum year (Gurung, Shah & Lamichhane 2019). The Lesotho Ante-Natal Care (LANC) Guidelines (Ministry of Health, Lesotho 2020) recommend that a pregnant woman has eight focused visits of which the initial visit occurs before or at 12 weeks gestational age and subsequent visits at 20, 26, 30, 34, 36, 38 and 40 weeks. Currently, the Lesotho Post-Natal Care (PNC) Standard Operating Procedures (SOPs) have no information on mental health or routine screenings that should be carried out. These SOPs do not recommend a PPD screening tool. On completion, PPD screening results are not attached to the obstetric record book. Therefore, women are discharged without being screened, diagnosed or managed for PPD, when present. In the Quthing district, there is only one hospital fully funded by the government. From the researcher’s work experience, there are no clear treatment and referral pathways for women with PPD following birth.

## Research methods and design

### Study design

This study adapted a qualitative, explorative, descriptive and contextual design. The constructivist paradigm was used for this study because it was seen as the most relevant avenue to explore and describe participant perceptions regarding PPD management. Furthermore, the constructivist paradigm was used as the researcher interacted with the participants with the belief that there are multiple realities of participants and with the assumption that knowledge is maximised when the distance between the inquirer and the participant in the study is minimised (Polit & Beck 2020). According to Brink, Van der Walt and Van Rensburg (2018), the constructivist paradigm integrates human interest into the study and assumes that access to reality is through social structures such as language, consciousness and shared meaning. Hence, data collection was focused on different realities experienced by participants.

### Study setting

The research study was conducted at Quthing Hospital, a government-funded healthcare facility situated in the southernmost district of Lesotho, known as Quthing. The hospital’s strategic location within the town of Moyeni, the district’s largest town, flanked by the Holy Trinity Anglican Church and the Lesotho Correctional Services facility, contextualises the research. The total number of nurse-midwives assigned to the maternity ward was 12; they covered triage, intrapartum, postpartum and newborn services, as needed. The average number of births each month was 900. This location provided a significant context for the research conducted in the study

### Study population and sampling strategy

In this study, purposive sampling was employed to intentionally select participants who met specific eligibility criteria. Out of 12 nurse-midwives working in the maternity ward, the researchers focused on those who had the relevant experience and qualifications necessary for the study. The nurse manager introduced the potential participants, and the researchers explained the study’s purpose. The selection process involved choosing nurse-midwives who had at least 2 years of experience, were employed full-time, were qualified to practice as professional midwives and had experience managing patients with PPD. Of the 12 midwives, 9 met these criteria. These nine participants were deliberately chosen because they were best positioned to provide valuable insights into the research topic, ensuring the data gathered would be rich and relevant.

### Data collection

The data-collection process took place between 27 August 2023 and 05 September 2023. Individual semi-structured interviews were conducted by the research assistant who was a nurse midwife, working at the non-governmental organisation (NGO). The researcher requested a research assistant to collect data to eliminate bias, as the researcher was a nurse midwife employed on the unit in question. The interviews were conducted in the Sesotho language as it was the common language of communication among the participants working within the hospital setting. Participants were individually interviewed to provide them with a unique opportunity to express themselves in a manner rarely available in their everyday lives. These participants were directly involved in the care of postpartum depressed patients. Each interview occurred in a private room during lunch time, typically lasted between 45 min and 60 min and was audio-recorded with the permission of the participants.

The research assistant collected insights from the perspective of the participants, employing open-ended questions to elicit responses. The primary semi-structured questions were: What are your perceptions regarding how PPD is addressed at the facility? What are your thoughts about how PPD is managed where the patients screen positive? How did your educational programme prepare you for screening, diagnosing and treating PPD? Interviews continued until data saturation was reached at the seventh participant when repetitive themes emerged. Two more interviews were conducted to confirm that no new information emerged. Table 1 details the primary research questions and prompts.

**TABLE 1:** Primary research questions and probes.

Primary research questions	Probes
What are your perceptions regarding how PPD is addressed at the facility?	How do you perceive PPD in your clinical practice? What specific signs or symptoms of PPD do you consider most significant or concerning? In what ways do you believe PPD affects the overall well-being of both the mother and the newborn?
What are your thoughts about how PPD is managed where the patients screens positive?	How do you currently approach the management of PPD in your clinical practice? Are there specific challenges or barriers that you face when implementing management strategies for PPD? How do you involve and educate the family in the management of PPD cases?
How did your educational programme prepare you for screening, diagnosing and treating PPD?	Are there instances where you find it difficult to maintain professional boundaries because of the emotional nature of PPD management? Do you feel adequately prepared or supported in terms of your own emotional well-being when managing PPD cases?

PPD, postpartum depression.

### Trustworthiness

Trustworthiness refers to the degree of confidence in the data, interpretation and methods used to ensure the quality of the study (Polit & Beck 2020). Credibility was maintained by the researcher engaging in the site with the participants, gaining familiarity and understanding of the context surrounding the persons being studied and asking participants to verify the researcher’s interpretations after transcribing the interviews. The information provided was verified by asking similar questions with all participants to see if their information was truthfully reflected. Dependability was enhanced by the researcher’s description of the steps taken and supporting them with literature reviews providing a clear understanding and describing every step before it was taken. Confirmability was maintained where the findings reflect the participants’ voices and conditions of the inquiry, not the researcher’s biases, motivations or perspective (Polit & Beck 2020). During coding, the researcher and the supervisor served as co-coders to ensure that coded data were a true representation of the participants’ data. Transferability was ensured by the researcher describing the research process and data analysis in detail so that others can follow a similar research process in a similar context.

### Data analysis

Data analysis happened shortly after data collection began. All interviews were transcribed verbatim and coded by the principal investigator. The principal investigator took field notes on all responses from participants written on note pads by the research assistant. Maguire and Delahunt’s (2017) thematic analysis method was adopted to analyse the data. The method included six steps. Step 1: Becoming familiar with the data, whereby the researcher gained familiarity with the data through reading and re-reading the transcripts of all individual interviews. Step 2: Generating initial codes by generating the first codes from each participant. Coding, which lowers large amounts of data into small pieces of meaning, was then utilised to organise the data in a meaningful and systematic manner, with each segment of data that was related to or captured anything noteworthy about the issue being coded. Each transcript was coded thematically based on every text section that appeared to directly answer the study topic (the supervisor re-read the section and re-considered if the code should be applied). New codes were generated as the researcher progressed through interview transcription. Step 3: Search for themes by reviewing the codes and compiling them into a topic and sorting into bigger themes that appeared to answer the study questions specifically. Step 4: Review themes, and step 5: Define themes by analysing what each topic was about, what it attempted to express and how it related to other themes. Step 6: Write up by constructing a final thematic map that demonstrated the relationship between themes (Maguire & Delahunt 2017). All the results were interpreted, backed and justified by the relevant literature. After data were analysed, the researcher and the researcher supervisor, who was an expert in the subject area of PPD, had a consensus meeting to discuss and agree on the identified themes and sub-themes.

### Ethical considerations

Ethical clearance for the study was granted by the Ministry of Health Research and Ethics Committee in Lesotho (reference no.: ID 197-2022) and the National University of Lesotho Institution Research Board (reference no.: NUL/MN8/2023/01). All procedures followed the ethical principles outlined in the Belmont report, including principles of beneficence, human dignity and justice. Permission to conduct the study was also granted by the Management of the Quthing District Hospital as well as the Nursing Manager of the maternity unit. The study aimed to benefit both individual participants and the society as a whole. Anonymity and confidentiality were assured through use of codes, ensuring that participant names remained undisclosed. Confidentiality was also maintained by encrypting transcripts and keeping materials in a locked room accessible only to the principal investigator and research supervisor. All data-collection materials will be disposed after 5 years of completion of the study. Written materials will be shredded and information stored electronically will be deleted. The principle of respect was upheld by providing participants with information about the study and their right to freely choose to participate and the right to withdraw without bias. Written informed consent was obtained from those who voluntarily agreed to participate, signifying their autonomy and respect for their rights.

## Results

### Demographics

A total of nine participants were interviewed and most were female. Participants ranged in age from 30 to 42 years. The majority of the participants held a Diploma in Nursing and Midwifery. Midwifery clinical experience ranged from 3 years to 15 years. Table 2 details demographic data related to participants.

**TABLE 2:** Demographic profile of participants (*N* = 9).

Gender	Age (years)	Participant number	Years of midwifery experience	Highest level of education
Female	32	1	12	Degree in nursing and midwifery
Female	42	2	8	Diploma in nursing and midwifery
Female	30	3	8	Diploma in nursing and midwifery
Female	36	4	7	Degree in nursing and midwifery
Female	30	5	4	Diploma in nursing and midwifery
Female	40	6	15	Diploma in nursing and midwifery
Male	33	7	4	Diploma in nursing and midwifery
Male	37	8	3	Diploma in nursing and midwifery
Male	41	9	3	Diploma in nursing and midwifery

### Themes and sub-themes

Three themes with eight sub-themes were identified on analysis. Themes included: a lack of nurse midwifery empowerment, inadequate human and material resources and stigma on mental health issues. Table 3 details the three major themes and corresponding sub-themes.

**TABLE 3:** Themes and sub-themes.

Themes		Sub-themes
1.	A lack of nurse midwifery empowerment	1.1 Management skills for PPD 1.2 Continuous professional development and training
2.	Inadequate human and material resources	2.1 Shortage of nurse-midwives 2.2 Shortage of guidelines, recording and reporting tools 2.3 Poor follow-up mechanisms
3.	Stigma on mental health issues	3.1 Self-perceived stigma 3.2 Stigma attached to PPD (community)

PPD, postpartum depression.

#### Theme 1: A lack of nurse midwifery empowerment

Participants perceived empowerment of nurse-midwives as vital in the effective management of PPD. Nurse-midwives felt they had inadequate information about PPD in their basic educational programme and that they did not receive adequate continuing professional education, which jeopardised the quality of PPD treatment. Participants expressed feelings of inadequate skill related to diagnosis and treatment of PPD, and continuing education was deemed insufficient.

**Sub-theme 1.1: Management skills for postpartum depression:** The lack of skill in the assessment and treatment of PPD was a perceived barrier in effective management of PPD. All participants expressed a lack of skill in the assessment, diagnosis and treatment of PPD, including psychotherapy interventions such as talk therapy:

‘We do not have skills to assess and manage PPD, we just rely on the discharge plan for the patient.’ (Participant 2, female, 8 years’ experience)

‘We have lack of knowledge and skills regarding managing PPD and during our clinical practice when we were nursing students, PPD was not in our procedure manual or register.’ (Participant 5, female, 4 years’ experience)

**Sub-theme 1.2: Continuous professional development and training:** Nurse-midwives viewed continuous professional development regarding PPD as crucial. Continuous professional development was deemed an in-service training programme that was provided by management to help develop and improve skills, which can be through refresher courses. Participants reported that continuous professional development enabled nurse-midwives to continually update and renew their knowledge and skills regarding PPD. Most of the participants verbalised a lack of training on PPD management in their clinical setting:

‘We do case presentation as part of our professional development, so think if we focus on cases like PPD, we can do better in its management, and again ownership of work can help a lot.’ (Participant 3, female, 8 years’ experience)

‘I believe we need trainings … continuous trainings in the hospital and practice regarding assessment and management of PPD …’ (Participant 9, male, 3 years’ experience)

#### Theme 2: Inadequate human and material resources

Nurse-midwives elucidated structural challenges around PPD management. They explained that the Lesotho Obstetric Record, Ante-natal Care Registers and PNC Registers do not include maternal mental health assessment, hence, proper management of PPD was incomplete, which limits the care needed. Participants also viewed shortage of nurse-midwives in the maternity ward as compromising the quality of care provided to the newly delivered mother.

**Sub-theme 2.1: Shortage of nurse-midwives:** Participants explained that a shortage of nurse-midwives had a negative impact on the management of PPD. Participants reported that low numbers of nurse-midwives were allocated to the maternity ward. High numbers of births and postpartum patients increased their work loads and impedes provision of quality care:

‘I think if our maternity ward can be well staffed, our allocation may put nurse-midwives in the delivery room, other in ante-natal care while other in post-natal care. In that way, complication can be easily identified. As it is now, there is a shortage of nurse-midwives.’ (Participant 1, female, 12 years’ experience)

‘There is shortage of nurse-midwives in maternity wards … we have a lot of clients but the problem is shortage of nurse-midwives, therefore we focus on delivery and on the infant and move as quick as possible to another client, forgetting conditions like PPD.’ (Participant 3, female, 8 years’ experience)

**Sub-theme 2.2: Shortage of guidelines, recording and reporting tools:** The majority of nurse-midwives expressed the shortage of guidelines, recording and reporting tools in managing PPD. Participants viewed guidelines as crucial in providing clinical decisions regarding PPD assessment and management, as well as rules of operation. Nurse-midwives also reported a lack of screening and reporting tools for PPD cases. Nurse-midwives explained there was a lack of information written on registers of women with PPD and how the lack of reporting tools impact on identifying and managing PPD, hence the severity of the condition was missed:

‘I think one major thing is there are no policies or guidelines on management of PPD, even the ante-natal registers, post-natal and Lesotho Obstetric record book has limited data on PPD screening, recording or reporting and its management.’ (Participant 1, female, 12 years’ experience)

‘There are no Standard Operating Procedures on assessment and … management of PPD, therefore, each and every nurse midwife manages clients with PPD there way he or she feels fit.’ (Participant 2, female, 8 years’ experience)

**Sub-theme 2.3: Poor follow-up mechanisms:** Participants perceived that one of the most challenging aspects of nurse-midwives is the fact that PPD is not prioritised as a serious condition. They also reported limited time to support women with PPD and poor follow-up when the PPD woman was discharged:

‘Post-delivery, we spend a short time with the women, so even the identified ones get lost, it’s like we need to do home visits to continue with management and link the women with village health care workers.’ (Participant 2, female, 8 years’ experience)

‘We don’t do follow-up’s … we totally don’t follow clients when at home, I think … I think the village healthcare workers need to be informed of such cases, so that they follow PPD women after delivery.’ (Participant 1, female, 12 years’ experience)

#### Theme 3: Stigma on mental health issues

Nurse-midwives noticed that there was often a negative attitude or stigma towards maternal mental issues with social disapproval for those with such problems. There was a need for PPD awareness to ensure the healthcare needs of postpartum women were met.

**Sub-theme 3.1: Self-perceived stigma:** Nurse-midwives perceived that there is often a perceived stigma regarding personal circumstances, which may have contributed to development of PPD. Such self-perceived stigma was viewed by participants as a person’s recognition that the community or society hold prejudice and discriminate against young women or girls who fall pregnant:

‘I think young women or girls already judge themselves that they felt pregnant at an early age or before marriage, which lead them to struggle to express how they feel.’ (Participant 3, female, 8 years’ experience)

‘Most young girls who deliver at the young age struggle to accept that they are now mothers because of our culture and tradition that young girls need to be married before marriage and at a certain age, now they already judge themselves while still in hospital after delivery.’ (Participant 7, male, 4 years’ experience)

**Sub-theme 3.2: Stigma attached to postpartum depression (community):** Participants explained how community stigma contributed to the postpartum woman having PPD. For example, adolescent mothers frequently encountered pregnancy-related stigma from community members, which increased their risk of PPD. Similarly, a pregnancy when reproductively older (age 45 or beyond) tended to be viewed negatively by community members:

‘In our society, teenage pregnancy is forbidden and when a teenager is pregnant, everyone around wants to know who is the father and that is depressing to the woman given their different circumstances.’ (Participant 3, female, 8 years’ experience)

‘Because of our culture and tradition, there is a lot of stigma on teenage pregnancy and pregnancy of an unmarried women, therefore people in a community are having a lot of discrimination and stigma on those unmarried, which causes a lot of stress to the young mother.’ (Participant 6, female, 15 years’ experience)

## Discussion

The results of this study revealed clarity on nurse-midwives’ perceptions regarding management of PPD. Participants perceived a lack of nurse-midwives’ empowerment in managing patients with PPD, where they highlighted that empowerment of nurse-midwives could provide them with mental health nursing skills necessary to identify early signs of PPD and its management. This aligns with the work of Almutairi et al. (2023), where participants held that nurse-midwives’ empowerment through education can help them to provide mental care to postpartum women based on their needs and intervene to prevent further deterioration. In addition, Jannati, Farokhzadian and Ahmadian (2021) assert that the healthcare system needs healthcare providers who are experts in mental health to diagnose and treat women with PPD.

Participants verbalised the lack of skills in the management of PPD including psychotherapy interventions. This is in line with the study conducted in Sri Lanka, where nurse-midwives reported to have limited skills regarding PPD and the majority had knowledge deficits regarding risk factors and PPD detection using EPDS (Kumarasinghe et al. 2022). However, Coates and Foureur (2019) argued that midwives and student midwives can provide mental healthcare with positive outcomes in terms of both physical and mental health outcomes for women, evidenced by counselling interventions.

Nurse-midwives considered professional development on PPD as crucial, seeing it as an in-service training programme. Nevertheless, participants observed a deficiency in training skills specifically related to PPD within their clinical settings. This is in line with the study conducted by Brugha et al. (2016), as cited in Wang et al. (2022), which revealed that nurse-midwives perceived training in psychological nursing strategies to be crucial and complementary to their initial professional knowledge. In addition, Lee et al. (2019) stated that in nursing organisations, empowerment leads to positive changes in the attitudes and behaviours of organisational members by increasing the capacity of nurse-midwives and spreading their power.

Nurse-midwives perceived adequate human and material resources crucial in the management of PPD. They shed light on the lack of screening tools and emphasised the need to address this gap, highlighting the importance of tools and interventions to improve PPD screening practices. This aligns with the recommendation that early screening for depression, starting during pregnancy, is pivotal in identifying the risk of PPD at an earlier stage, facilitating timely interventions that can span the entire postpartum period (Almutairi et al. 2023). As Jannati et al. (2021) underscore, the absence of timely diagnosis and treatment for PPD carries multifaceted consequences, encompassing a diminished quality of life, strained maternal relationships, hindered infant growth and development, marital discord and even the emergence of suicidal ideation. Participants reported low numbers of nurse-midwives allocated to the maternity ward increase their workloads and tamper with provision of quality care. This is similar to the study conducted by Bayrampour et al. (2018), cited in Castello (2021), which found understaffing as provider- and system-level barriers to inadequate screening of PPD in the obstetrical setting. This is also in line with the study conducted by Setebe and Kiwara (2022), which reported that the barriers for nurse-midwives’ performance included inadequate staffing level and increased workloads. McCauley et al. (2022) stated that the introduction of routine mental health guidelines and standardised questionnaires help guide the nurses in the formulation of registers and the assessment of women during routine ante-natal and post-natal contact.

In this study, nurse-midwives highlighted the challenges surrounding the management of PPD, including a lack of healthcare policies and guidelines, reporting tools, information in the registers and staff shortages, all of which impact the identification and management of PPD in healthcare settings. This is in line with the study conducted by Almutairi et al. (2023), which asserts that policies and procedures must be updated on a regular basis to reflect the latest evidence-based practice.

Furthermore, participants showed the perceived challenge of poor follow-up mechanisms, particularly with regard to community engagement involving village health workers, to identify maternal depression, facilitate successful referrals and alleviate PPD symptoms. This aligns with the findings by Kallem et al. (2019), where a very low rate of home visits for postpartum mothers to screen for PPD was uncovered. Similarly, most of the nurses in the study of Almutairi et al. (2023) stated that postpartum women who are at risk of or have depression need to be followed up and monitored utilising home visits, which focus on maternal mental health. It was indicated that because of the risk of PPD developing at any time, home care should be considered a solution for following up on the mental health status of postpartum women. They also reported limited time to support women with PPD and poor follow-up when the PPD woman was discharged. The study conducted in the United Kingdom by Ford et al. (2019) found that 42% of women who screened positive for PPD did not see their General Doctor for support, citing the logistical challenges of access to nurse-midwives and doctors as primary reasons for not attending appointments.

In summary, participants shed light on the significant role of stigma, with a focus on the need to empower the family and the influence of self-perceived stigma, in predisposing clients to PPD. Similarly, participants in the research by Almutairi et al. (2023) emphasised that feelings of guilt and stigma deter postpartum women from seeking help for depression. They recommended addressing negative attitudes towards mental health and raising awareness to facilitate timely healthcare access for postpartum women.

Nurse-midwives perceived that there is often a perceived stigma regarding personal circumstances, which may have contributed to development of PPD. This is consistent with Dubrieucq et al. (2021) who conveyed that perceived and experienced stigma may lead one to develop mental illness and there is need to develop anti-stigma and recovery-oriented campaigns. In addition, the study conducted by Sakina et al. (2022) revealed that the majority of participants did not share their deteriorating health and symptoms with anyone, because they thought that their social and cultural setting is such that people might not understand and they present various justifications about it.

Participants explained how community stigma contributed to the postpartum woman having PPD. This finding agrees with the study conducted in Ethiopia by Monaghan et al. (2021), where women were socially expected to be strong and symptoms of depression such as sadness and crying were regarded as weakness and religious sin. It is reported that in the hospital, families are generally receptive to an early nurse home visit, particularly if they have previously experienced home visiting (Handler et al. 2019). Participants explained the importance of family involvement in the care of the woman post-delivery and the need to inform caregivers and spouses about the presence or increased risk of PPD. This is supported by a study that stated that family members who have a good level of education and have come across women with PPD, hold positive attitudes towards PPD, hence health education to family members is important (Poreddi et al. 2021).

### Strengths and limitations

This study provided a comprehensive exploration of the perceptions of nurse-midwives regarding PPD educational preparation, care of such patients in the clinical setting and the need for ongoing professional continuing education. Findings offered in-depth insights into the experiences and perspectives regarding PPD when nurse-midwives were providing care in a busy in-patient setting. Utilising qualitative research methodology such as interviews, allowed for a rich exploration of subjective experiences, enabling a nuanced understanding of the topic. The study was conducted exclusively in Quthing district, which may limit the generalisability of the findings to other districts in Lesotho. As the healthcare infrastructure, resources and cultural factors might differ across regions, the perceptions gathered in Quthing district may not fully represent those of nurse-midwives or patients in other parts of the country.

### Recommendations

Further research is crucial including other districts of Lesotho, using both qualitative and quantitative research approaches. Recommendations that emanated from the participants accounts is to empower nurse-midwives through the continuous professional development to enhance their competencies on PPD assessment, diagnosis and its management.

## Conclusion

Nurse-midwives’ perception of their education on PPD indicates a deficiency in empowerment, inadequate human and material resources and the existence of stigma. This perception regarding PPD education was evident among all participating nurse-midwives in the study. Consequently, it is imperative for hospital management to offer continuous support to nurse-midwives, as this will lead to positive outcomes in the maternity ward.
